# Prevalence of JC Virus in Chinese Patients with Colorectal Cancer

**DOI:** 10.1371/journal.pone.0035900

**Published:** 2012-05-11

**Authors:** Xiaozhou Mou, Ling Chen, Fanlong Liu, Jian Lin, Pingping Diao, Haohao Wang, Yifei Li, Jianjiang Lin, Lisong Teng, Charlie Xiang

**Affiliations:** 1 State Key Laboratory for Diagnosis and Treatment of Infectious Diseases, the First Affiliated Hospital, Zhejiang University School of Medicine, Hangzhou, China; 2 Department of Oncology, the First Affiliated Hospital, School of Medicine, Zhejiang University, Hangzhou, China; 3 Institute of Molecular Pathology and Program in Molecular Cell Biology, Zhejiang University School of Medicine, Hangzhou, China; 4 Molecular Diagnosis Division, Zhejiang-California International Nanosystems Institute (ZCNI), Hangzhou, China; The University of Hong Kong, Hong Kong

## Abstract

**Background:**

JCV is a DNA polyomavirus very well adapted to humans. Although JCV DNA has been detected in colorectal cancers (CRC), the association between JCV and CRC remains controversial. In China, the presence of JCV infection in CRC patients has not been reported. Here, we investigated JCV infection and viral DNA load in Chinese CRC patients and to determine whether the JCV DNA in peripheral blood (PB) can be used as a diagnostic marker for JCV-related CRC.

**Methodology/Principal Findings:**

Tumor tissues, non-cancerous tumor-adjacent tissues and PB samples were collected from 137 CRC patients. In addition, 80 normal colorectal tissue samples from patients without CRC and PB samples from 100 healthy volunteers were also harvested as controls. JCV DNA was detected by nested PCR and glass slide-based dot blotting. Viral DNA load of positive samples were determined by quantitative real-time PCR. JCV DNA was detected in 40.9% (56/137) of CRC tissues at a viral load of 49.1 to 10.3×10^4^ copies/µg DNA. Thirty-four (24.5%) non-cancerous colorectal tissues (192.9 to 4.4×10^3^ copies/µg DNA) and 25 (18.2%) PB samples (81.3 to 4.9×10^3^ copies/µg DNA) from CRC patients were positive for JCV. Tumor tissues had higher levels of JCV than non-cancerous tissues (*P* = 0.003) or PB samples (*P*<0.001). No correlation between the presence of JCV and demographic or medical characteristics was observed. The JCV prevalence in PB samples was significantly associated with the JCV status in tissue samples (*P*<0.001). Eleven (13.8%) normal colorectal tissues and seven (7.0%) PB samples from healthy donors were positive for JCV.

**Conclusions/Significance:**

JCV infection is frequently present in colorectal tumor tissues of CRC patients. Although the association between JCV presence in PB samples and JCV status in tissue samples was identified in this study, whether PB JCV detection can serve as a marker for JCV status of CRC requires further study.

## Introduction

The John Cunningham virus (JCV) is a ubiquitous, small, non-enveloped polyomavirus with a closed, circular, double-stranded DNA genome that frequently resides in the kidneys of healthy individuals and is excreted in the urine of a large portion of the adult population. JCV infection is subclinical and leads to lifelong latency, but may be reactivated when the immune system is impaired [1]–[3]. JCV is associated with disease primarily in immunocompromised individuals and its replication in glial cells could lead to progressive multifocal leukoencephalopathy (PML), an often fatal disease of central nervous system [4]. Like other polyomaviruses, JCV encodes a version of a large T-antigen that can bind to and inactivate tumor suppressor proteins p53 and pRB and interfere with several cell-signaling pathways [5]–[7]. Moreover, JCV genomic DNA sequences and T-antigen expression have been detected in a broad range of human tumor cell types including oligodendrocytes, astrocytomas, glioblastomas, ependymomas, and most other types of brain tumors [8], indicating that JCV infection may be associated with human carcinogenesis.

More recently, JCV was also found in non-neural cancers, such as gastric [9] and lung cancers [10]. JCV infection was first reported as a potential risk factor for colorectal cancer (CRC) in a work by Laghi et al. [11], which found that 96% of CRC tissues were positive for JCV DNA sequences. Subsequently, other studies have been published showing that JCV sequences were found in 26%–88.9% of CRC tissues, with sample sizes ranging from 18 to 105 [12]–[15]. In contrast, other studies have subsequently shown that no detectable JCV DNA was present in colorectal tissues indicating no association between JCV and colon neoplasia [16]–[17]. JCV prevalence in various CRC sample sets may be due to differences in assay sensitivities across studies, contamination between samples, or geographic locations of the selected population. Although demonstration of the presence of the tumor viral components is the first step to characterize the role of JCV in CRC carcinogenesis, additional evidence, such as high viral load, is necessary to support a causal role. However, only two studies have mentioned the viral load of JCV in CRC [11], [15]. Considering the controversial reports on the etiology of JCV in CRC, it appears that the association between JCV infection and CRC needs to be further investigated.

Many studies have demonstrated that JCV DNA or antigens can be detected in the peripheral blood (PB) or serum. Viral components detected in the blood have historically been presumed to have originated from metastasized cancer cells or from virus-containing cellular debris shed from a local infection site [18]. Although two prospective case-control studies have been performed to determine the association between JCV seropositivity and CRC development [19]–[20], there has been no report describing the presence of JCV DNA in the blood of CRC patients published to date. In this study, we investigated the prevalence of JCV in Chinese CRC patients to determine a potential relationship. In addition, we investigated whether JCV DNA in PB is suitable as a diagnostic marker for JCV-related CRC.

## Methods

### Ethics statement

This project was approved by the Ethics Committee of the First Affiliated Hospital, College of Medicine, Zhejiang University, Hangzhou, China. Written informed consent was provided by patients and controls.

### Clinical samples

All clinical samples were obtained between May 2007 and October 2011 from patients admitted to the First Affiliated Hospital, College of Medicine, Zhejiang University (Hangzhou, China). Tumor tissues, non-cancerous tumor-adjacent tissues and PB samples from 137 random CRC patients (mean patient age, 62.99; range, 27–86 years) were collected less than three months after CRC diagnosis. Patients undergoing surgery for recurrences were excluded. Non-cancerous tumor-adjacent tissue was collected from an area ∼15 cm distal from the tumor. In addition, 80 colorectal tissues from non-pathologic mucosa of patients undergoing colonoscopy for evaluation of functional disorders such as diarrhea or constipation unrelated to cancer or inflammatory bowel disease (IBD) and 100 PB samples from healthy donors upon physical examination with no history of cancer or polyp diagnosis were collected as controls. Tissue samples were divided; one part was kept for histopathological analysis, and the other transferred to cryotubes, immediately frozen in liquid nitrogen, and stored at −196°C for further analysis. All sample data were recorded in a sample collection database.

### DNA extraction

About 50 mg of each tissue sample was homogenized in a 2 ml sterile tube with 1 ml of 0.09% sodium chloride solution using an electric homogenizer (PRO Scientific, Oxford, CT, USA). To minimize cross-contamination between samples, the probe was washed in fresh sterile water three times, 40% hydrogen peroxide solution twice, 75% ethanol twice and heated in a 95% ethanol burner for 3 min between samples. Four hundred microliters of each PB sample was used for DNA isolation. The DNA of each sample was extracted using the MasterPure DNA Purification Kit (Epicentre Biotechnologies, Madison, WI, USA) according to the manufacturer's instruction. To confirm the integrity of DNA extracted, PCR using glyceraldehydes phosphate dehydrogenase (GAPDH) primers was undertaken as described previously [21].

### Nested PCR for the JCV genome

Nested PCR amplification was performed using a set of primers for T-antigen as described previously [13]. Briefly, the 173 bp target of JCV T-antigen was first amplified by using of the primers JCT-1F (5′-AGT CTT TAG GGT CTT CTA-3′) and JCT-1R (5′-GGT GCC AAC CTA TGG AAC AG-3′). First round PCR condition was denatured at 95°C for 10 min, followed by 30 cycles of denaturation at 95°C for 15 s, annealing at 55°C for 30 s and extension at 72°C for 30 s. Nested PCR was performed by using 2 µl of the first-round product as a template, with the primers JCT-1F and JCT-2R (5′-TGA AGA CCT GTT TTG CCA TG-3′). The nested PCR condition was denatured at 95°C for 10 min, followed by 30 cycles of denaturation at 95°C for 15 s, annealing at 60°C for 30 s and extension at 72°C for 30 s. The nested PCR procedure amplified a 110 bp target sequence. To avoid potential PCR false-positive results, two water controls were included for first round PCR and nested PCR. If either control of the two water controls yielded a false-positive in the nested PCR, the whole set of PCR and nested PCR was restarted with new reagent. For each sample, 1 µl of DNA was amplified by nested PCR. Four microliters of each amplification products were analyzed by electrophoresis on 2% agarose gel and ethidium bromide staining.

### Glass slide-based dot blotting

The nested-PCR products were purified with MinElute PCR Purification Kit (QIAGEN, Hilton, Germany) and finally eluted in 10 µl EB buffer (10 mM Tris-Cl, pH 8.5). Purified DNA samples were mixed with one volume of DMSO, and spotted onto surfaces of aminosilane slides (CapitalBio, Beijing, China) as three replicates with a SmartArrayer spotter (CapitalBio, Beijing, China). After baking for 1 h at 80°C, the slides were rinsed in 0.2% SDS for 5 min and cleaned by washing with ddH_2_O. TAMRA-labeled oligonucleotide probe (JCT-probe, 5′-GTT GGG ATC CTG TGT TTT CAT CAT C-3′) was purchased from Invitrogen (Shanghai, China) and diluted to a concentration of 10 µM. Hybridization was carried out at 45°C for 2 h. The slides were washed in 2×SSC/0.2% SDS at 42°C for 5 min, and finally in ddH_2_O at 42°C for 5 min. The slides were dried by centrifugation and scanned with a GenePix 4100 scanner (Axon, Union, USA). Images were analyzed with GenePix Pro 5.0 software (Axon).

### Quantitation of viral load by real-time PCR

A SYBR Green real-time PCR method was used to determine JCV viral load in samples as described by Chapagain [22]. Specially, primers RT-JCT-F (5′-AGA GTG TTG GGA TCC TGT GTT TT-3′) and RT-JCT-R (5′-GAG AAG TGG GAT GAA GAC CTG TTT-3′) were used in the real-time PCR targeting a sequence of T-antigen gene, producing a 78 bp product during reaction. JCV viral load quantitation was performed in the Bio-Rad CFX96 Real-time PCR Detection System using 200 ng of template DNA, 10 µl of SYBR Green PCR Master Mix (TaKaRa, Dalian, China) and 4 pmol each of forward and reverse primers. Thermal cycling was initiated with a first denaturation step at 95°C for 30 s, followed by 40 cycles of 95°C for 5 s and 60°C for 15 s and the amplification fluorescence were monitored at 60°C at the end of each cycle. A standard curve was constructed using serial dilutions (1.0 to 10^7^ copies of JCV DNA) of JCV T-antigen plasmid. Three replicates were performed for each sample and real-time PCR data were analyzed using Bio-Rad CFX manager software. The averaged copy numbers in samples were calculated according to the standard curve and were represent as copies of viral DNA per microgram genomic DNA.

### Statistical methods

Demographic characteristics were compared between CRC patients (C) and controls (non-CRC patients (N) or healthy donors (H)) using the *Student's t*-test for numeric variables and the χ^2^ test for categorical variables. Sex, tobacco exposure, alcohol use, residence, family history of CRC, pathogenic stage and tumor site were compared between JCV-positive and -negative CRC-patients using the χ^2^ test. Differentiation status was compared between JCV-positive and -negative CRC patients using the Fisher's exact test. The exact logistic regression model, adjusted for age, was used to estimate the association between JCV infection and CRC. R statistical software (v 2.12.0, www.r-project.org/) was used for statistical analysis. In all analyses, a *P*-value<0.05 was considered statistically significant.

## Results

Demographic characteristics of 137 CRC patients, 80 non-CRC patients and 100 healthy donors are present in Table 1. CRC patients were older than non-CRC patients (*P*<0.001), but no difference was observed between CRC patients and healthy donors. Family history of CRC was more common among CRC patients verses non-CRC patients or healthy donors, but the differences were not statistically significant (*P* = 0.491 for C vs N, *P* = 0.162 for C vs H).

**Table 1:** Demographic characteristics of CRC patients (C), non-CRC patients (N) and healthy donors (H).

Characteristic	CRC patients n = 137	Non-CRC patients n = 80	Healthy donors n = 100	*P* C vs N	*P* C vs H
Age (mean ± SD)	63.0±12.6	49.6±13.3	62.6±13.1	<0.001	0.817
Sex				0.940	0.985
Male	74 (54.0)^c^	44 (55.0)	56 (56.0)		
Female	63 (46.0)	36 (45.0)	44 (44.0)		
Tobacco exposure^a^				0.922	0.998
Yes	44 (32.1)	27 (33.8)	33 (33.0)		
No	93 (67.9)	53 (66.2)	67 (67.0)		
Alcohol use^b^				0.785	0.859
Yes	29 (21.2)	19 (23.8)	23 (23.0)		
No	108 (78.8)	61 (76.2)	77 (77.0)		
Residence				0.7557	0.511
City	71 (51.8)	44 (55.0)	57 (57.0)		
Country	66 (48.2)	36 (45.0)	43 (43.0)		
Family history of CRC				0.491	0.162
Yes	27 (19.7)	12 (15.0)	11 (11.0)		
No	110 (80.3)	68 (85.0)	89 (89.0)		

a , No tobacco exposure was defined as never having smoked cigarettes daily for more than 1 year.

b , No alcohol use was defined as never having consumed 1 drink or more per month.

c , Values within parentheses indicate percentages.

**Figure 1:**
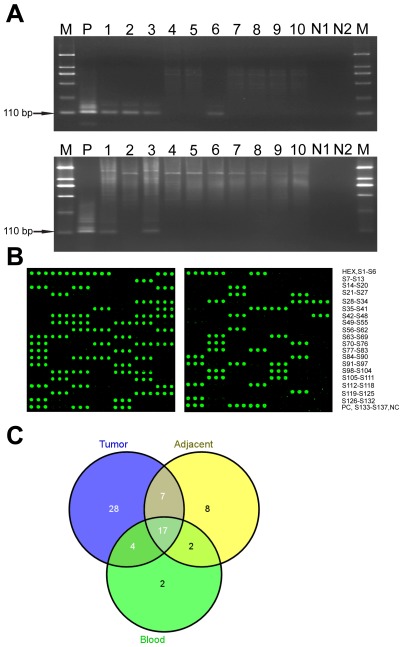
Detection of the JCV T-antigen sequence in samples from CRC patients. (A) The 110 bp fragment was amplified from DNA isolated from matched samples of colorectal cancer (upper) and normal tumor adjacent tissues (lower) with nested PCR. M: DL 2,000 DNA Marker (TaKaRa); P: positive control; N1: first-round negative control; N2: second-round negative control. (B) Images from JCV nested-PCR product arrays. Nested-PCR products of tumor tissues (left) and normal tumor adjacent tissues (right) were spotted onto surfaces of aminosilane slides as three replicates, hybridized with TAMRA-labeled oligonucleotide probe and finally visualized by AXON scanner. (C) The numbers of JCV-positive samples in 137 matched pairs of tumor tissue, non-cancerous adjacent tumor tissue and PB samples from CRC patients.

All samples were positive for the GAPDH control, indicating adequate integrity of the samples. The negative controls in both rounds of PCR revealed no evidence of reagent contamination. Using nested PCR, the JCV T-antigen nucleotide sequence was detected in 40.9% (56 of 137) of tumor tissues and 24.8% (34 of 137) of tumor-adjacent tissues from CRC patients (Figure 1). The PCR products were confirmed as authentic JCV sequences by glass-slide array analysis (Figure 1B). We found both matched pairs of tumor tissue and non-cancerous tissue from 17.5% (24 of 137) of CRC patients were positive for JCV DNA. 23.4% (32 of 137) and 7.3% (10 of 137) of patients had JCV DNA only in tumor tissue or non-cancerous tumor-adjacent tissue, respectively (Figure 1C). 18.2% (25 of 137) of PB samples from CRC patients were positive for JCV DNA. A significantly higher JCV presence was observed in CRC tissues compared with non-cancerous adjacent tissues (OR, 2.089; 95% CI, 1.213–3.636; *P* = 0.007) and PB samples (OR, 3.084; 95% CI, 1.729–5.619; *P*<0.001). JCV presence in PB samples was positively associated with JCV status in tissue samples (*P*<0.001); however, it was unrelated to patient age, sex, tobacco exposure, alcohol use, residence, family history of CRC, pathologic stage and tumor site but tended to increase with the differentiation of CRC tissues, although this trend was not statistical significant (Table 2). Compared with tissues from CRC patients, only 13.8% (11 of 80) of colorectal tissues from non-CRC patients had detectable JCV DNA. In the non-parameter statistical analysis, JCV frequencies in CRC patients and non-CRC patients were significantly different. Since the age of the two groups differed, the association between JCV infection and CRC was estimated by exact logistic regression analysis, adjusted for age. The OR of JCV associated CRC risk was statistically significant (OR, 6.611; 95% CI, 2.928–14.929; *P*<0.001). Among the PB samples from 100 healthy volunteers, we found that only 7% (7 of 100) were positive for JCV DNA, which was lower than the JCV positive percentage in PB samples from CRC patients (OR, 0.339; 95% CI, 0.118–0.852; *P* = 0.021).

**Table 2:** Factors associated with JCV infection among CRC patients.

Characteristic	JCV-positive n (%)	JCV-negative n (%)	*P*
Age (mean ± SD)	64.2±13.0	61.8±12.3	0.261
Sex			0.438
Male	39 (53.4)	34 (46.6)	
Female	29 (45.3)	35 (54.7)	
Tobacco exposure^a^			0.330
Yes	25 (56.8)	19 (43.2)	
No	43 (46.2)	50 (53.8)	
Alcohol use^b^			0.708
Yes	13 (44.8)	16 (55.2)	
No	55 (50.9)	53 (49.1)	
Residence			0.929
City	36 (50.7)	35 (49.3)	
Country	32 (48.5)	34 (51.5)	
Family history of CRC			0.183
Yes	17 (63.0)	10 (37.0)	
No	51 (46.4)	59 (53.6)	
Chemotherapy^d^			0.213
Yes	13 (65)	7 (35)	
No	55 (47.0)	62 (53)	
Pathogenic stage			0.807
I, II	39 (48.1)	42 (51.9)	
III, IV	29 (51.8)	27 (48.2)	
Differentiation			0.477^c^
Poor	26 (53.1)	23 (46.9)	
Moderate	39 (50.0)	39 (50.0)	
Well	3 (30.0)	7 (70.0)	
Tumor site			0.887
Rectum	42 (48.3)	45 (51.7)	
Left colon	11 (50.0)	11 (50.0)	
Right colon	15 (53.6)	13 (46.4)	

a , No tobacco exposure was defined as never having smoked cigarettes daily for more than 1 year.

b , No alcohol use was defined as never having consumed 1 drink or more per month.

c , by Fisher's exact test. d, Chemotherapy was used before surgery within 3 months.

**Figure 2:**
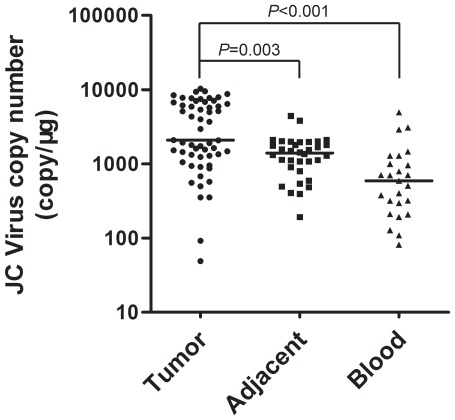
JCV viral load was higher in tumor tissues than in non-cancerous tumor adjacent tissues and PB samples. JCV positive samples determined by nested PCR were analyzed using qRT-PCR. Blots represent averaged copy numbers (3 replicate wells) of JCV DNA for tumor tissue, non-cancerous tissue and PB, respectively, and bars represent calculated medians. *P*-values were calculated using the Wilcoxon's test.

Viral load in JCV-positive samples from CRC patients were determined by a quantitative real-time PCR (qRT-PCR). The viral load in tumor tissues ranged from 49.15 to 1.03×10^4^ copies/µg DNA (mean ± SD, 3795.64 ±3054.58). Actually, the absolute copy numbers for JCV DNA in CRC tumors were lower than 1 copy per cell. Low JCV viral load was observed in 34 JCV-positive, non-cancerous, tumor-adjacent tissues (range, 192.85 to 4.44×10^3^ copies/µg DNA; mean ± SD, 1470.09±887.12) and 25 PB samples (range, 81.30 to 4962.99 copies/µg DNA; mean ± SD, 945.17±1140.39). Tumor tissues had significantly higher levels of JCV DNA than non-cancerous tumor-adjacent tissues and PB samples (Figure 2).

## Discussion

In the present study, JCV was detected in 40.9% of CRC tissues and 24.8% of non-cancerous colorectal tissues, which were in the intermediate position of T-antigen rate in previous studies (Table 3). Several potential reasons account for the lack of agreement among these studies, including potential contamination during sample collection and process during DNA extraction and PCR amplification. Many studies did not report any step to control false-positive results [11]–[12], [14]. In the present study, we avoided false-positive results as mentioned in our previous study [21]. The sensitivity of the method is critical, as a low JCV viral load in colorectal tissue has been documented [15]. Immunohistochemistry, PCR, nested PCR and q-RT PCR have been used for detecting JCV [11], [14]–[15], [23]. JCV DNA was detected in 1% and 0% of CRC tissues in two large studies [16]–[17] using a general PCR assay, which is known to have lower sensitivity. Nested PCR is a highly sensitive technique for detecting JCV. Smaller PCR targets have been found to be more readily amplified than long sequences for JCV detection [11]. As described by Hori et al. [13], we used a nested PCR with primer sets targeting a 110 bp JCV T-antigen sequence, which was able to detect as few as 1 copy of JCV DNA (data not shown). It has been hypothesized the supercoiled topology of the highly homologous JCV DNA might limit PCR amplification efficiency [11]. However, we did not use topoisomerase I treatment before PCR amplification as described by Laghi et al. [11], which may lead to a slightly lower rate of JCV infection. Age, gender, region and lifestyle of a selected population could all contribute to varying rates of JCV infection (Table 3). The American CRC population was most commonly selected in previous studies, in which JCV DNA was found in 0%–96% of CRC tissues [11], [14], [16]–[17], [24]. Contradictory results have been reported by two Italian research groups [12], [25]. Hori et al. [13] and Lin et al. [23] identified JCV in 26.1% and 86.4%, respectively, of CRC tissues from Asian populations. In our study, JCV DNA was detected in 49.6% of Chinese CRC patients.

**Table 3:** Summary of the findings of JC virus studies in CRC patients.

Author and reference citation	Country	Design	Positive cases/total cases	Prevalence (%)	Sample source	Detecting method	Primers or region amplified
Laghi et al. [11]	USA	Case	23/24	96	Fresh tissue	Nested PCR	T-antigen
Casini et al. [12]	Italy	Case	15/18	83.3	Paraffin-embedded tissue	Southern blot, PCR, ISH	T-antigen
Enam et al. [14]	USA	Case	22/27	81.4	Paraffin-embedded tissue	PCR	T-antigen, agnoprotein
Goel et al. [24]	USA	Case	77/100	77	Paraffin-embedded tissue	PCR	T-antigen
Hori et al. [13]	Japan	Case-control	6/23	26.1	Paraffin-embedded tissue	Nested PCR	T-antigen, VP, agnoprotein
Lin et al. [23]	Taiwan	Case	19/22	86.4	Paraffin-embedded tissue	Nested PCR	T-antigen
Campello et al. [25]	Italy	Case-control	0/94	0	Not known	qRT-PCR	T-antigen
Theodoropoulos et al. [15]	Greece	Case-control	49/80	61.3	Paraffin-embedded tissue and fresh tissue	Nested PCR, qRT-PCR	T-antigen
Newcomb et al. [16]	USA	Case	0/233	0	Paraffin-embedded tissue	PCR, qRT-PCR	T-antigen, VP1, RR
Hernandez et al. [17]	USA	Case	1/100	1	Paraffin-embedded tissue	PCR	T-antigen
This study	China	Case-control	47/98	48.0	Fresh tissue	Nested PCR	T-antigen

PCR, polymerase chain reaction; qRT-PCR: quantitative real-time PCR. ISH, in situ hybridization; VP, virus capsid protein; RR, regulator region.

We further investigated the presence of JCV DNA in tissue from non-CRC patients. In certain high-risk patients, such as those with polyp or IBD, the incidence of CRC is substantially higher. Therefore, patients undergoing colonoscopy for polyp and IBD were excluded from the control cohort before sample collection. Other than age, there was no baseline characteristic significantly different between CRC and non-CRC patients. However, adjustment for age did not change the association between JCV infection and CRC. Combining with the observation of higher prevalence of JCV in tumor tissue from CRC patients, we propose that JCV is correlated with CRC and may participate in CRC carcinogenesis or, alternatively, the virus infects tumor cells more readily than non-cancerous cells.

In the present study, no correlation between the presence of JCV and demographic or medical characteristics such as age, gender, education, clinical stage, and tumor site was observed, which was consistent with other studies [13], [15]. Interestingly, we found that tissues from CRC patients who received chemotherapy prior to sample collection had higher incidence of JCV infection compared with the patients without chemotherapy, although the difference was not significant (Table 2). The association between immunosuppression and increased susceptibility to infection is well recognized. As demonstrated by Selgrad et al. [26] in a retrospective study among liver transplant recipients, Immunosuppressed patients have a significantly higher presence of JCV compared with immunocompetent controls. Chemotherapy drugs can inhibit the immune response and enable the reactivation of potentially oncogenic viruses. CRC patients with a family history have a higher prevalence of JCV T-Ag DNA, which is consistent with the results described by Vilkin et al. [27]. JCV infection is usually an asymptomatic infection and commonly occurs in later childhood and adolescence, after which the virus remains latent in the kidney. Besides the immune condition mentioned above, genetic background might also be associated with the susceptibility of JCV infection in CRC.

The viral load in tissue samples could indicate the role of JCV in CRC carcinogenesis. In the present study, JCV viral load in positive samples from CRC patients were determined by q-RT PCR. JCV viral copy numbers were found to be higher in tumor tissues as compared with non-cancerous tumor adjacent tissues, which is consistent with previous studies [11], [15]. However, the absolute copy numbers in tissues were so low that only a small part of colorectal epithelial cells were found to be infected with JCV. This result supports the transient effects of JCV in cellular transformation, as it can be silenced or its genome can be lost during cancer progression (“hit-and-run” transformation) [28]. A JCV infection introducing the T-antigen could easily explain the onset of chromosomal instability in multistep carcinogenesis, such as that previously proposed for the well-known adenoma-carcinoma sequence [29].

To the best of our knowledge, this study is the first analysis of JCV DNA in PB from CRC patients. We showed that JCV infection is common in PB samples from CRC patients and indicated JCV status in tissue samples. Unlike previous seroepidemiological studies, our results cannot be used to predict the risk of CRC because all samples were collected within three months of diagnosis [19]–[20]. Furthermore, the presence of JCV in PB of CRC patients was higher than in PB of healthy donors. These results supported the proposition that JCV DNA in PB samples could be a valid biomarker for JCV-related CRC diagnosis. However, medical characteristics of healthy donors were recorded mainly through self-report which may lead to misclassification. More importantly, JCV almost always remains latent in the kidney and can be easily detected in urine [1]–[3]. Although the viral DNA in bloodstream is widely considered to originate from cells shed from tumor tissue, the origin of the JCV in PB samples remains unknown.

In conclusion, JCV infection presents commonly in CRC patients and might to be a risk factor for CRC. We suggest ongoing molecular, cellular and *in vivo* studies to elucidate the mechanisms of JCV carcinogenesis to determine whether JCV is a causative agent of CRC. Although we found JCV DNA in PB samples could serve as a biomarker for JCV-related CRC, further large-scale population-based studies are encouraged to evaluate this association.
